# The Terrestrial Biosphere Model Farm

**DOI:** 10.1029/2021MS002676

**Published:** 2022-02-16

**Authors:** Joshua B. Fisher, Munish Sikka, Gary L. Block, Christopher R. Schwalm, Nicholas C. Parazoo, Hannah R. Kolus, Malen Sok, Audrey Wang, Anna Gagne‐Landmann, Shakirudeen Lawal, Alexandre Guillaume, Alyssa Poletti, Kevin M. Schaefer, Bassil El Masri, Peter E. Levy, Yaxing Wei, Michael C. Dietze, Deborah N. Huntzinger

**Affiliations:** ^1^ Jet Propulsion Laboratory California Institute of Technology Pasadena CA USA; ^2^ Schmid College of Science and Technology Chapman University Orange CA USA; ^3^ Woodwell Climate Research Center Falmouth MA USA; ^4^ National Snow and Ice Data Center Cooperative Institute for Research in Environmental Sciences University of Colorado Boulder CO USA; ^5^ Department of Earth and Environmental Sciences Murray State University Murray KY USA; ^6^ Centre for Ecology and Hydrology Penicuik UK; ^7^ Environmental Sciences Division Oak Ridge National Laboratory Climate Change Science Institute Oak Ridge TN USA; ^8^ Department of Earth and Environment Boston University Boston MA USA; ^9^ School of Earth and Sustainability Northern Arizona University Flagstaff AZ USA

**Keywords:** terrestrial biosphere model, land surface model, vegetation model, ecosystem model, Earth System Model, ecoinformatic, model intercomparison project, PEcAn

## Abstract

Model Intercomparison Projects (MIPs) are fundamental to our understanding of how the land surface responds to changes in climate. However, MIPs are challenging to conduct, requiring the organization of multiple, decentralized modeling teams throughout the world running common protocols. We explored centralizing these models on a single supercomputing system. We ran nine offline terrestrial biosphere models through the Terrestrial Biosphere Model Farm: CABLE, CENTURY, HyLand, ISAM, JULES, LPJ‐GUESS, ORCHIDEE, SiB‐3, and SiB‐CASA. All models were wrapped in a software framework driven with common forcing data, spin‐up, and run protocols specified by the Multi‐scale Synthesis and Terrestrial Model Intercomparison Project (MsTMIP) for years 1901–2100. We ran more than a dozen model experiments. We identify three major benefits and three major challenges. The benefits include: (a) processing multiple models through a MIP is relatively straightforward, (b) MIP protocols are run consistently across models, which may reduce some model output variability, and (c) unique multimodel experiments can provide novel output for analysis. The challenges are: (a) technological demand is large, particularly for data and output storage and transfer; (b) model versions lag those from the core model development teams; and (c) there is still a need for intellectual input from the core model development teams for insight into model results. A merger with the open‐source, cloud‐based Predictive Ecosystem Analyzer (PEcAn) ecoinformatics system may be a path forward to overcoming these challenges.

## Key Points

- We ran nine terrestrial biosphere models centralized on a common computing framework
- The Farm allows multiple MIP experiments to be run relatively quickly and uniformly
- Challenges included technological demand, model versioning, and interpretation of results

## 1 Introduction

**Figure 1:**
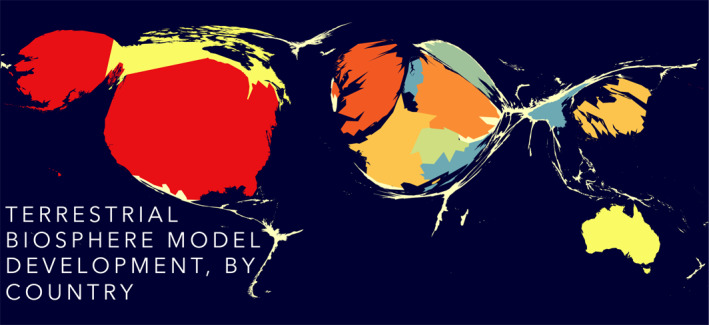
Most global terrestrial biosphere models (TBMs) are developed primarily in the US, Europe, Australia, and Japan. This cartogram inflates/deflates the size of the country based on the number of TBMs developed, *n* = 58. From Fisher et al. (2014).

Models are among our best integrators of knowledge and understanding of how the world works. Terrestrial biosphere models (TBMs) encapsulate a myriad of land surface and ecosystem processes, and how those respond to one another and external forcings (Fisher et al., 2014). But, knowledge is biased and socially constructed (Berger & Luckmann, 1966; Nietzsche, 1977); consequently, we too may expect our models to be locally biased to the construction of knowledge that created them. How photosynthesis is understood to someone in the US may be different than that to someone in Brazil or Australia or Japan (Rogers et al., 2017). Still, global models must be constructed for global science, though their construction is predominantly local (Figure 1).

The great "elephant in the room," so to speak, is not what is behind us, but what is ahead. Projections of the future hold no truth in the present, yet are best read by how well we see in the past. Like the fable of the blind men and the elephant, we want to know what is in front of us—the future of the Earth—that which we cannot see. Each model and modeler, like the blind men, is biased to their local knowledge, each saying something different, yet similar. These model differences define the uncertainty of our future (IPCC, 2014). By working together, we may piece together a more accurate picture than in isolation.

Model Intercomparison Projects (MIPs) are that “working together.” MIPs are designed to hear different perspectives to sample the population of possible ways the world works. In theory, if that sample is sufficiently robust, then one might converge toward a central tendency of likely truth. Ensemble model means typically perform better than most individual models (Schwalm et al., 2015). The distribution of multimodel variability could be used to construct "emergent constraints" for benchmarks to base future projections (Bretherton & Caldwell, 2020; Brient, 2020; Cox et al., 2018; Eyring et al., 2019; Hall et al., 2019; Nijsse et al., 2020; Tokarska et al., 2020). In practice, however, that sampling is often not robust and likely skewed toward models that are derivatives of one another, thereby compounding local biases (Caldwell et al., 2018; Huntzinger et al., 2013, 2017; Sanderson et al., 2015, 2021). Still, MIPs are a necessary starting point for a conversation; otherwise, it is more like a lot of voices shouting in dissonance.

Due to numerous user decisions, the same model code run 10 times can have 10 different outputs. If our interest is in understanding variability in biophysical processes (Bonan & Doney, 2018), then this is a problem, as there should, in theory, be no process representation variability for a single model (with notable exceptions, e.g., Lawrence et al., 2019; Niu et al., 2011). What could potentially cause different outputs from the same model and can we control for it? Starting with the starting point or spin‐up—a model should move forward from the same initial conditions every time, but this can easily vary depending on how one wants to create the world (Huntzinger et al., 2020; Shi et al., 2013). The forcing data used could vary, for example, from one meteorological data set to another, even if the model stays the same (Badgley et al., 2015; Lawrence et al., 2019). How a model/modeler uses the same forcing data could vary, for example, mapping of model plant functional types (PFTs) onto a land cover/land use change (LCLUC) data set (e.g., “mixed forest,” different crops). A model might have functions that can be turned on or off and may be reliant on availability of certain forcing data (e.g., nitrogen cycle). Finally, the output may all be identical, but the interpretation of the output can vary from person to person or be altered in transfer or analysis.

Now, compound this all with multiple different models, and we can see how comparing models for variability in process representation can quickly become muddled by other factors that can influence model output. MIPs attempt to control for many of these factors, but struggle to ensure that their protocols are rigorously met. For TBMs alone, there have been a lot of MIPs (global, regional, site; offline, coupled), and there will be more—e.g., PILPS (Henderson‐Sellers et al., 1993), VEMAP (VEMAP, 1995), CCMLP (Kicklighter et al., 1999), NPP MIP (Cramer et al., 1999), DGVM MIP (Cramer et al., 2001), C4MIP (Friedlingstein et al., 2006), AMMA (Redelsperger et al., 2006), GSWP (Dirmeyer et al., 2006), WETCHIMP (Melton et al., 2013), LBA‐DMIP (de Gonçalves et al., 2013), NACP Interim Site and Regional Syntheses (Huntzinger et al., 2012; Schwalm et al., 2010), TRENDY (Sitch et al., 2013), MsTMIP (Huntzinger et al., 2013), ISI‐MIP (Warszawski et al., 2014), FACE MDS (Medlyn et al., 2015), CMIP (Arora et al., 2020), and PalEON (Rollinson et al., 2021) (see Table 1 for acronyms and abbreviations). MIPs typically specify common forcing data, but some models need the data transformed (e.g., temporally) or require additional individualized data sets. Some MIPs specify spin‐up criteria, but those are not necessarily applicable across all models (Huntzinger et al., 2013). Finally, a model might adhere strictly to the MIP protocol, but some element of tuning to common benchmark data occurs such that inter‐model output converges, despite differences in processes (Dommenget & Rezny, 2018; Fer et al., 2021; Hourdin et al., 2017; Notz, 2015; Raäisaänen, 2007; Scheiter et al., 2013).

**Table 1:** Acronyms and Abbreviations

Acronym/Abbreviation	Definition
AMMA	African Monsoon Multidisciplinary Analysis
API	Application Programming Interface
C4MIP	Coupled Climate Carbon Cycle Model Intercomparison Project
CABLE	CSIRO Atmosphere Biosphere Land Exchange
CAM	Community Atmosphere Model
CASA	Carnegie‐Ames‐Stanford Approach
CCMLP	Carbon‐Cycle Model Linkage Project
CESM	Community Earth System Model
CLASS‐CTEM‐N	Canadian LAnd Surface Scheme—Canadian Terrestrial Ecosystem Model—Nitrogen
CLM	Community Land Model
CMIP	Coupled Model Intercomparison Project
CMS	Carbon Monitoring System
DGVM	Dynamic Global Vegetation Model
DLEM	Dynamic Land Ecosystem Model
ESM	Earth System Model
FACE MDS	Free Air CO_2_ Enrichment Model Data Synthesis
GFDL‐CM3	Geophysical Fluid Dynamics Laboratory—Coupled Model
GSWP	Global Soil Wetness Project
GTEC	Global Terrestrial Ecosystem Carbon
HadGEM2‐AO	Hadley Center Global Environment Model version 2—Atmosphere‐Ocean
I/O	Input/Output
IPSL‐CM5A‐MR	Institut Pierre Simon Laplace—Coupled Model—Mid‐Resolution
ISAM	Integrated Science Assessment Model
ISI‐MIP	Inter‐Sectoral Impact—Model Intercomparison Project
JPL	Jet Propulsion Laboratory
JULES	Joint UK Land Environment Simulator
LBA‐DMIP	Large‐Scale Biosphere‐Atmosphere—Data Model Intercomparison Project
LCLUC	Land Cover Land Use Change
LPJ‐GUESS	Lund‐Potsdam‐Jena—General Ecosystem Simulator
LPJ‐wsl	Lund‐Potsdam‐Jena—Wald Schnee und Landschaft
LSCE	Laboratoire des Sciences du Climat et de l'Environnement
LUH‐SYNMAP	Land Use Harmonization—Synergetic Land Cover
MIP	Model Intercomparison Project
MPI‐ESM‐MR	Max Planck Institute—Earth System Model—Mixed Resolution
MsTMIP	Multi‐scale Synthesis and Terrestrial Model Intercomparison Project
NACP	North American Carbon Program
NASA	National Aeronautics and Space Administration
NetCDF	Network Common Data Form
NPP	Net Primary Productivity
ORCHIDEE	ORganizing Carbon and Hydrology In Dynamic Ecosystems Environment
PalEON	Paleo‐Ecological Observatory Network
PEcAn	Predictive Ecosystem Analyzer
PFT	Plant Functional Type
PILPS	Project for Intercomparison of Land Surface Parameterization Schemes
RCP	Representative Concentration Pathways
SG3	Simulation Global 3
SiB	Simple Biosphere
TBM	Terrestrial Biosphere Model
TEM	Terrestrial Ecosystem Model
VEGAS	VEgetation Global Atmosphere & Soil
VEMAP	Vegetation‐Ecosystem Modeling & Analysis Project
VIC	Variable Infiltration Capacity
VISIT	Vegetation Integrative SImulator for Trace gases
WETCHIMP	Wetland CH4 Intercomparison of Models Project

As a means to address some of these outstanding issues in MIPs, we sought to develop a prototype system whereby models are run with common operating software on the same hardware systems (Fer et al., 2021; Kumar et al., 2006). This approach is similar to multimodel systems, such as the Land Information System (LIS; Kumar et al., 2006), the Terrestrial Observation and Prediction System (TOPS; Nemani et al., 2002), and the Predictive Ecosystem Analyzer (PEcAn; Dietze et al., 2013). Such a system ensures that spin‐up protocols are run consistently across models, user decisions on forcing data such as LCLUC‐to‐PFT mapping are systematic, there is no tuning, and that output is saved, transferred, and analyzed uniformly. Further, logistical coordination among multiple teams can be streamlined. We focused on global TBMs and used the relatively strict MsTMIP protocol for spin‐up and factorial model experiments from 1901 to 2010 (Phase I) and 2011 to 2100 (Phase II; Huntzinger et al., 2013). Overcoming two major challenges was required for this to be successful: (a) development of a software system that could interface consistently across disparate models as well as supercomputing infrastructure and (b) compilation and setup of multiple models. Here, we describe this system, called the Terrestrial Biosphere Model Farm, named with tribute to a similar initial effort by others (Denning et al., 2009). The Model Farm conceptually builds on efforts such as LIS with hydrological strengths, but here with more of an emphasis on terrestrial carbon cycling. We show an example output for illustration, but the focus of this paper is descriptive rather than output analytical. Finally, we provide experiential lessons‐learned and recommended paths forward.

## 2 Methods

### 2.1 Data and Models

We primarily used the MsTMIP data and simulation protocols (Huntzinger et al., 2013; Wei et al., 2014). Phase I consisted of simulations from years 1801 to 2010 and Phase II from 2011 to 2100, all globally at 0.5° × 0.5° resolution. Phase I forcing data included climate, LCLUC, atmospheric CO_2_, and nitrogen deposition. These drivers were turned on/off (i.e., variable or constant) semi‐factorially such that the influence of each driver can be isolated in analysis (Huntzinger et al., 2013). Spin‐up to initial conditions was dictated by steady‐state criteria (Huntzinger et al., 2013). Climate variables were given at a 6‐hourly temporal resolution derived from a CRU‐NCEP merged product (Harris et al., 2014; Kalnay et al., 1996). LCLUC was derived from a merged LUH‐SYNMAP product, resulting in 47 classes annually (Hurtt et al., 2011; Jung et al., 2006). Monthly atmospheric CO_2_ was constructed for consistency with GLOBALVIEW among other databases (Wei et al., 2014). Finally, annual nitrogen deposition was constructed based on Dentener et al. (2006).

MsTMIP Phase II simulations were forced with climate and CO_2_ projections from five CMIP5 Earth System Models (ESMs) and two Representative Concentration Pathways (RCPs). The ESMs included CESM1‐CAM5 (Meehl et al., 2013), GFDL‐CM3 (Griffies et al., 2011), HadGEM2‐AO (Bellouin et al., 2011), IPSL‐CM5A‐MR (Dufresne et al., 2013), and MPI‐ESM‐MR (Giorgetta et al., 2013). The ESMs span the full range of model climate sensitivity between low‐ and high‐temperature response. The RCPs included business‐as‐usual (RCP8.5) and medium mitigation scenarios (RCP4.5) (Van Vuuren et al., 2011). LCLUC was held constant, and nitrogen deposition was not used in our Phase II simulations, though they are available as time‐varying to 2100.

The TBMs used in the Farm included CABLE (Wang et al., 2010), CENTURY (Parton et al., 1988), HyLand (Levy et al., 2004), ISAM (El‐Masri et al., 2013), JULES (Clark et al., 2011), LPJ‐GUESS (Smith et al., 2001), ORCHIDEE (Krinner et al., 2005), SiB‐3 (Baker et al., 2008), and SiB‐CASA (Schaefer et al., 2008). We also used the output from the MsTMIP version 1 models for comparison (Huntzinger et al., 2013), which included: CLASS‐CTEM‐N (S. Huang et al., 2011), CLM4 (Bonan et al., 1995), CLM4VIC (Lei et al., 2014), DLEM (Tian et al., 2012), GTEC (Ricciuto et al., 2011), ISAM (Jain & Yang, 2005), LPJ‐wsl (Sitch et al., 2003), ORCHIDEE‐LSCE (Krinner et al., 2005), SiB‐CASA (Schaefer et al., 2008), TEM6 (Hayes et al., 2011), VEGAS2.1 (Zeng et al., 2005), and VISIT (Ito, 2010). We do not describe the models in detail here, instead referring the reader to the respective references. Different models were run for different combinations of simulations and model experiments with all completing spin‐up, some completing some or all of the MsTMIP Phase I experiments, and some completing all of the MsTMIP Phase I and II experiments (see Section 3), depending on application (see, e.g., Discussion).

### 2.2 Model Farm

The Terrestrial Biosphere Model Farm was designed to keep individual TBM codes intact and treated as modular or compartmentalized, which can also facilitate the replacement of models by new versions. Each TBM is wrapped in the Farm software infrastructure, interfacing with the original model codes without modification, enabling different forcing data sets to be used, and unifying model output into defined formats.

**Figure 2:**
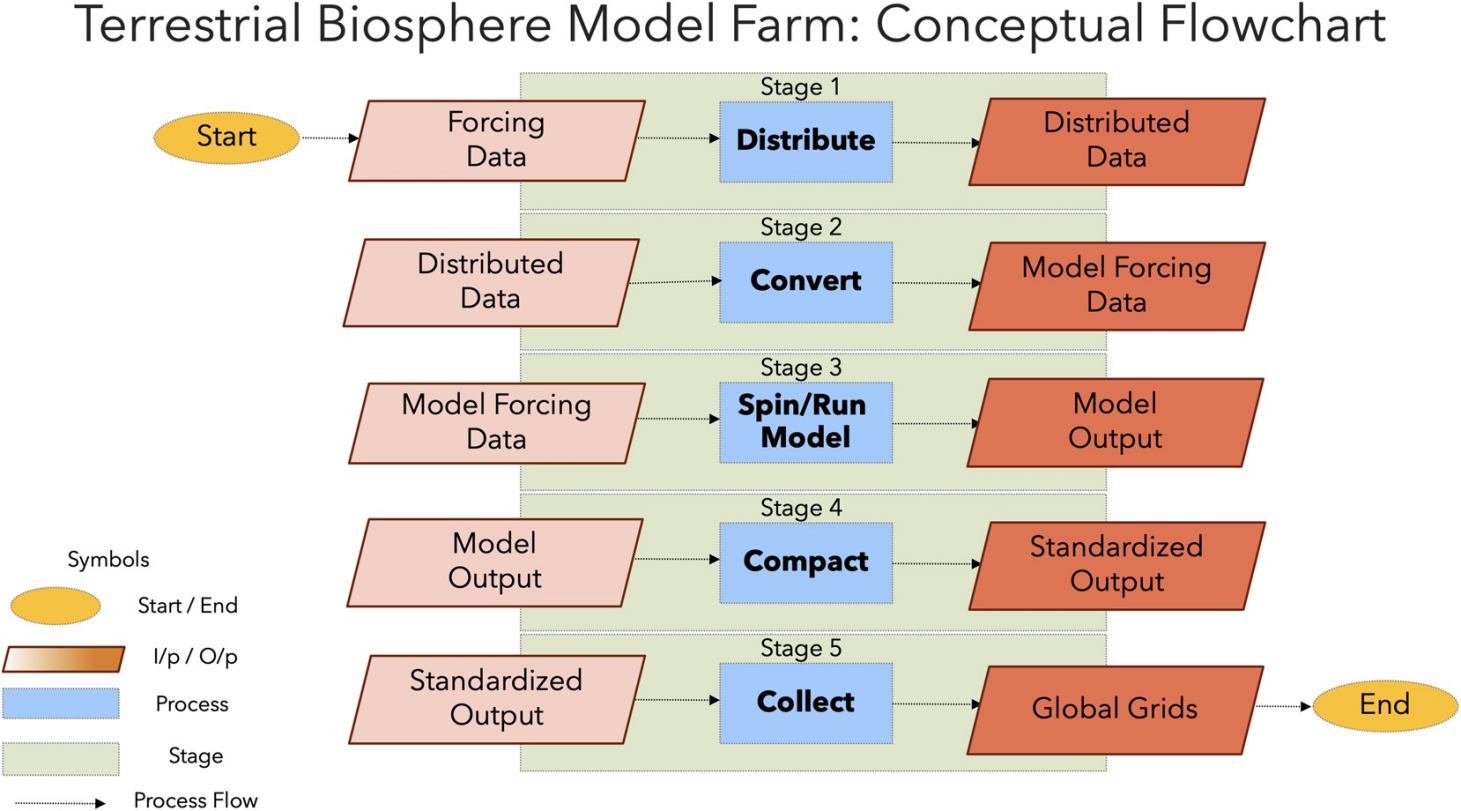
Terrestrial Biosphere Model Farm software flowchart consists of five stages. Stage 1: forcing data (e.g., from MsTMIP) are partitioned and distributed among supercomputer nodes. Stage 2: forcing data are converted to individual model requirements (format, temporal resolution, and units). Stage 3: models are spun up and run. Stage 4: model output is converted again (format, temporal resolution, and units) for standardization across models. Stage 5: model output is gathered off supercomputing nodes and recombined into global (or otherwise) grids.

The Terrestrial Biosphere Model Farm consists of five stages from distribution of forcing data to collection of model output (Figure 2): (a) Data Distribution, (b) Data Conversion, (c) Spin/Run Model, (d) Compact Output, and (e) Collect Output. Finally, a Diagnostics package enables automatic and relatively immediate visualization of model output to check for problems and/or success. The Model Farm was written in a combination of C++, Fortran, Matlab, and R for different functions. The Model Farm migrated over three different NASA JPL supercomputers (Zodiac, Halo, and Gattaca) during the course of development due to infrastructure upgrades (Table 2).

**Table 2:** High‐Performance Computing Specifications for the Terrestrial Biosphere Model Farm Project Generally Improved as the Project Migrated Across NASA JPL Supercomputers From Zodiac to Halo to Gattaca

System name	Zodiac	Halo	Gattaca
Project block quota	30 TB	10 TB	15 TB/high‐performance scratch filesystem^a^
Project file # (inode)	1 million	10 million	9 million (scratch_lg^b^)|4 million (scratch_sm^b^)
Interconnect (I/O)	Dual 40 Gb/s^b^	40 Gb/s^b^	100 Gb/s^b^
Total cores	1,920	1,328/1,040	4,608
Number of cores/node	12	16	48
Memory/node	24 (2 GB/core)	64 (4 GB/core)	384 (8 GB/core)
Local scratch	None	1 TB HDD/node	365 GB SSD/node|1.9 TB HDD/node

^a^ *n* = 4.

^b^ InfiniBand.

#### 2.2.1 Stage 1: Data Distribution

Data Distribution restructures all input files into the supercomputing environment. This is the most time‐consuming of all the stages, but is done only once per forcing data set, and used for all models. Here, the global forcing data are read, split into "patches" (i.e., groups) of cells, and distributed onto supercomputing nodes for parallel processing such that each patch has an approximately similar computational load. This split can be done because there is no lateral/horizontal cell‐to‐cell transport (e.g., water, carbon, and energy) for these models. Some patches may contain more grid cells than others due to empty ocean cells (i.e., land‐only data). The global grid from MsTMIP was a 1‐degree spatial resolution at 181 × 360 latitude and longitude or 65,160 cells (Wei et al., 2014). We distributed these data into 2,000 patches, each with ∼32 cells (or "points") per patch, on 2,000 matching supercomputing processors/nodes based on memory and storage capacities of the nodes (with modifications as we migrated supercomputers). An index file assigns a patch and point number for each grid cell along with a time dimension, also forcing each model to operate with the same spatial resolution. This index file may be matched to index files from new data sets that need to align in space and time, for example, connecting historical data (1901–2010; Phase I) to future projections (2011–2100; Phase II), which may have disparate grid sizes and north/south orientations. MD5 checksums ensure that no data are corrupted in transfer from one computer to another.

The time required to complete Data Distribution varies depending on hardware infrastructure. Initially, the Data Distribution stage took 3–4 months to complete, but eventually came down to 3–4 days to complete per data set as we migrated across supercomputers. The bottleneck in the first supercomputer, Zodiac, was due to file transfer Input/Output (I/O) capacity and file count capacity, which were later expanded in the subsequent supercomputers, Halo and Gattaca. While I/O was expanded sufficiently in Halo, our project storage was reduced (from 30 TB in Zodiac to 10 TB in Halo), which required additional time to manage. MsTMIP forcing data required >500 GB (split roughly equally between Phase I and II). Finally, in Gattaca, the I/O and storage were sufficient for efficient Data Distribution.

#### 2.2.2 Stage 2: Data Conversion

Data Conversion restructures the input forcing data to meet the unique requirements of individual models. Stage 2 is applied to each model after the model is successfully compiled and tested with provided test data or single point data on the supercomputer. Stage 2 ensures that there are no changes to the model code within the Farm. Specifically, forcing data may be converted to different: (a) formats such as NetCDF 3 or 4, ascii, *.mat, etc.; (b) temporal resolutions (e.g., hourly and daily); and (c) units, depending on the model. LCLUC is assigned to model PFTs. Configuration parameters are generated for each model to specify which data sets to access. This control module can be updated to swap out different forcing data sets for different domains and experiments. Spin‐up data may be reorganized for length of runs. Time required for data conversion was approximately 6 hr per data set transformation. Additional storage was required for the converted data (e.g., >2 TB for SiB3). After Stage 2 is complete, a model may be ready to spin‐up and run various global simulations.

#### 2.2.3 Stage 3: Spin and Run Model

Spin and Run Model runs the model to meet defined steady‐state initial conditions and/or executes a desired transient model experiment. Here, independent instances of the model for each of the 2000 patches are scheduled for parallel execution. In theory, Stage 3 should run smoothly; in practice, however, several issues must be managed for complete success. In spin‐up, individual patches may fail to meet spin‐up requirements within the run time allotted and must be rerun (Shi et al., 2013). Incorrect or incongruent LCLUC assignments may cause a model to crash a patch due to unspecified model parameters for the assignment. Forcing data sets may not align with each other (e.g., LCLUC assignment, but no climate). An automated failed‐patch detector was written to rerun patches with longer run times to handle some spin‐up issues. Other failed patches had to be identified visually, for example, noticeable as stripes or unexpectedly high/low values in a global map or as spikes in diagnostic zonal/latitudinal line plots. These patches were then gathered by the Farm operator for rerun and/or debugging. For efficiency, the Model Farm framework allowed for individual patches to be rerun without requiring the entire global data set to be rerun. Spin‐up required 1–2 days for most patches to complete, depending on model and supercomputer. Transient run time also required 1–2 days, depending on model, supercomputer, and simulation (e.g., 100 years historical‐only vs. 200 years historical plus future).

#### 2.2.4 Stage 4: Compact Output

Compact Output converts output from individual models into common formats and units for analysis. Model output from Stage 3 is produced in individual model‐dependent formats, units, variables, and time steps. Stage 4 unifies model output across models. Here, we convert all model outputs into NetCDF, common units (e.g., as defined by MsTMIP), standardized NaN/water/ice values, and monthly time steps. The monthly time step conversion reduces storage and transfer time demand, as most models operate and output at sub‐monthly time steps (e.g., daily for HyLand and 6‐hourly for CABLE), and which gives Stage 4 its name. For example, pre‐Stage 4, HyLand used 1 TB per simulation; whereas, CABLE used nearly 4 TB per simulation. Monthly compaction requires approximately half a day (e.g., HyLand) to a day (e.g., CABLE) per simulation per model depending on the Stage 3 output. Converting output file formats (e.g., *.out files from CENTURY to NetCDF) requires approximately another day.

#### 2.2.5 Stage 5: Collect Output

Collect Output converts all the individual patches into a common global grid. The final stage in the Model Farm requires collecting all the outputs, which are still partitioned into patches on individual supercomputer nodes and pieces the patches back together into a cohesive and standardized global grid (still at the monthly time step from Stage 4). Metadata are added at this stage, including, for example, model source code, model version, and contact information. Model output files are organized as one variable per file per model. Stage 5 collection requires less than half a day per simulation, depending on the number of variables saved.

## 3 Results

**Figure 3:**
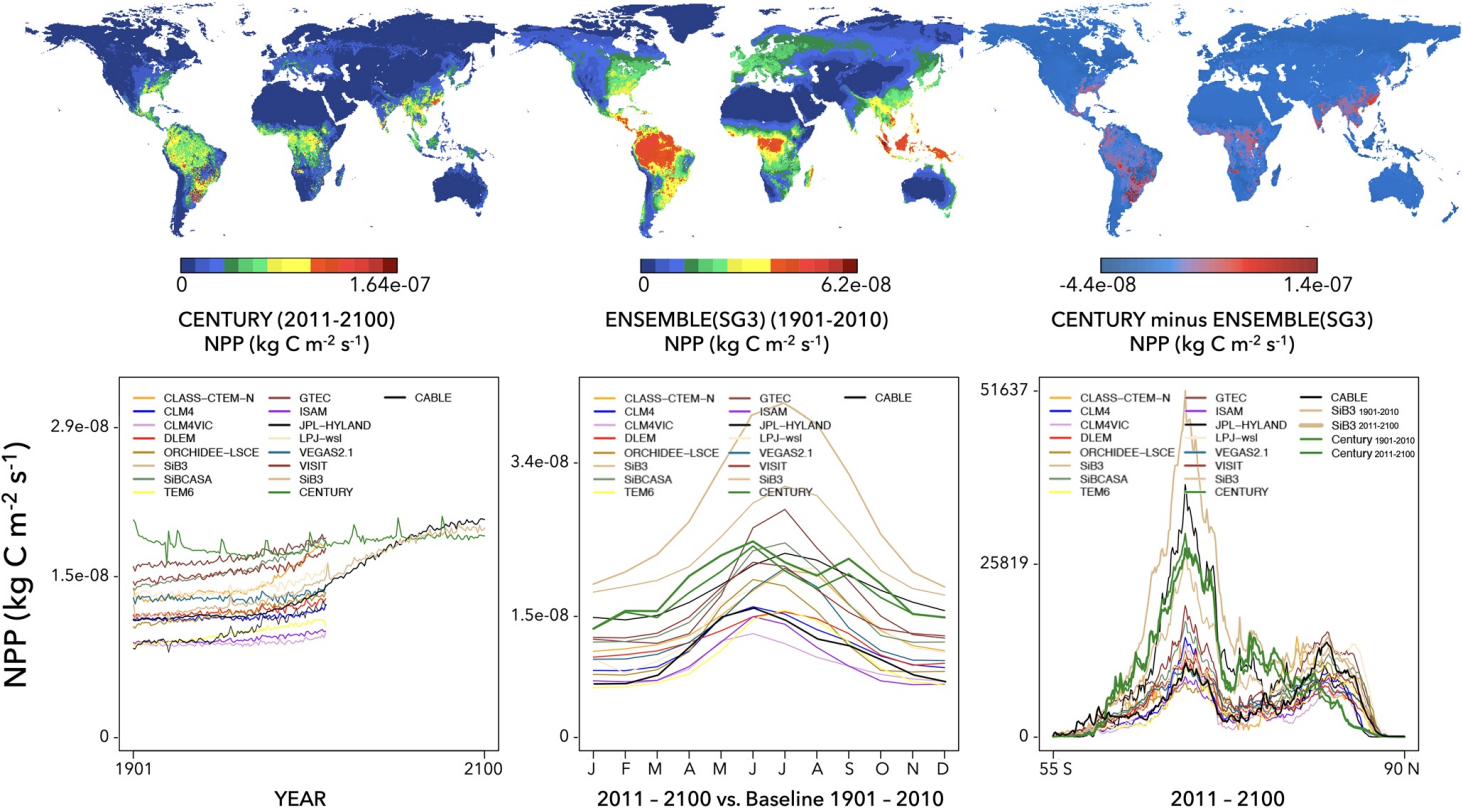
An automated diagnostic visualization PDF is generated from the Terrestrial Biosphere Model Farm. (top‐left) Map of average variable value over simulation time span; (top‐middle) comparison map of average variable value; (top‐right) difference map between 1 and 2; (bottom‐left) annual time series along with comparison models; (bottom‐middle) average monthly values with comparison models; and (bottom‐right) average zonal/latitudinal values with comparison models. Here, we show Net Primary Productivity (NPP) with CENTURY as the highlighted model of interest for the MsTMIP Phase II simulation (2011–2100) with CESM1‐CAM5 climate and RCP4.5 CO_2_ scenario. Comparison models are from MsTMIP Phase I SG3 simulation. Four additional models from the Farm are shown for 1901–2010 (CABLE, CENTURY, HyLand, and SiB‐3) with the latter three also shown for the Phase II simulation.

The Model Farm includes two visual diagnostic packages: the Single Variable package and the Experiment package. The Single Variable package is automatically run after collecting the model output from spin‐up or a transient run. The user repeats the Experiment package as needed. These packages allow the user to quickly evaluate the model output and identify any problems. The Single Variable package produces a single PDF page per variable per model for all output variables (Figure 3). Each page includes six panels, including three maps and three line plots: (a) map of average variable value over simulation time span, (b) map of average variable value over simulation time span from comparison models, (c) difference map between (a) and (b), (d) annual time series with comparison models, (e) average monthly values with comparison models, and (f) average zonal/latitudinal values with comparison models. A specific model of interest is given a thicker line than the other models for visualization (Purdy et al., 2019). Axis and map ranges are automatically set to minimum and maximum extent of data visualized. The user must develop their own customized packages to answer specific scientific questions and to alter and improve esthetic visualization of the model output. The Experiment package produces inter‐simulation comparisons. The Diagnostics package requires a few hours per variable to produce the plots for the global MsTMIP‐structured output.

An example of the Single Variable package visualization is shown in Figure 3. Here, we show Net Primary Productivity (NPP) for CENTURY as the highlighted model for the MsTMIP Phase II simulation (2011–2100) with CESM1‐CAM5 climate and RCP4.5 CO_2_ scenario. The top‐left map displays the 2011–2100 annual NPP. The top‐middle map shows the mean NPP from the MsTMIP Phase I models, SG3 simulation (CO_2_, climate, and LCLUC all "on"); we note that the 1901–2010 time period for SG3 is not an equivalent time comparison to the 2011–2100 runs, but it provides a first‐order check. The top‐right map shows the difference between the first two maps.

The bottom‐left panel of Figure 3 shows the annual time series from 1901 to 2100. Here, we can see that the MsTMIP Phase I SG3 simulations are given only from 1901 to 2010. Additional Model Farm output is shown for 1901–2010 from CABLE, CENTURY, HyLand, and SiB‐3. This figure also shows Model Farm Phase II simulations for 2011–2100 for CENTURY, HyLand, and SiB‐3. The bottom‐middle panel shows the mean seasonal cycle for all models; two lines are given each to CENTURY, HyLand, and SiB‐3, one line for 1901–2010 and the other for 2011–2100. Finally, the bottom‐right panel shows the zonal/latitudinal averages again with two lines each for the three Phase II models. These six figures combined are useful diagnostics to determine if there any obvious problems with the model runs. For example, a unit conversion error (e.g., Stage 2) can lead to model output significantly outside the spread of the other models.

**Figure 4:**
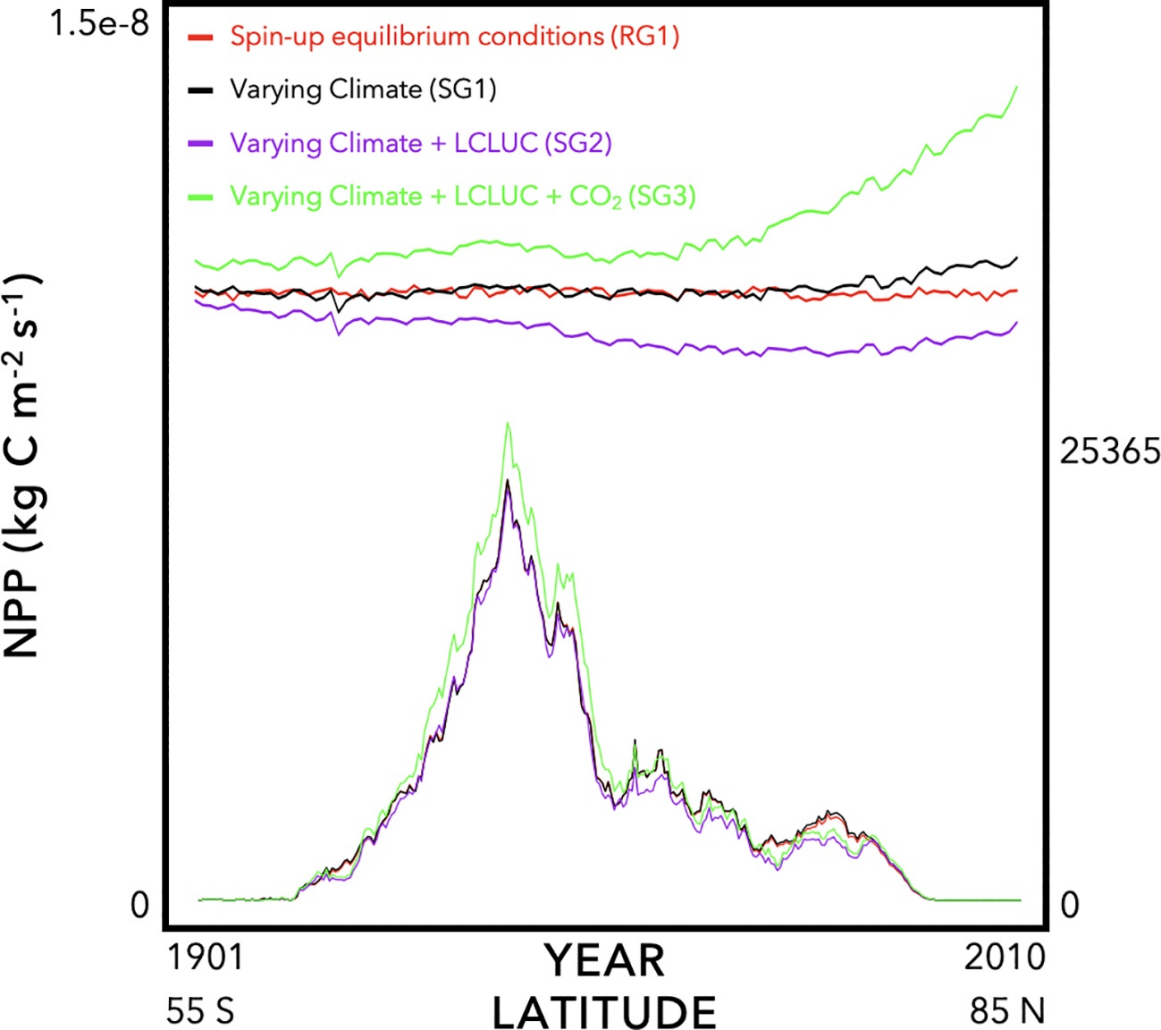
Inter‐simulation visual diagnostics for MsTMIP Phase I experiments show how a single model changes output among different experiments. Here, we show output (e.g., Net Primary Productivity, NPP, for HyLand) from four experiments: (1) spin‐up equilibrium conditions (RG1); (2) varying climate (SG1); (3) varying climate and land use/land cover change (LCLUC) (SG2); and (4) varying climate, LCLUC, and CO_2_ (SG3). The top set of lines (primary *y*‐axis) is the annual average from 1901 to 2010. The bottom set of lines (secondary *y*‐axis) is the latitudinal/zonal sum from 55°S to 85°N.

**Figure 5:**
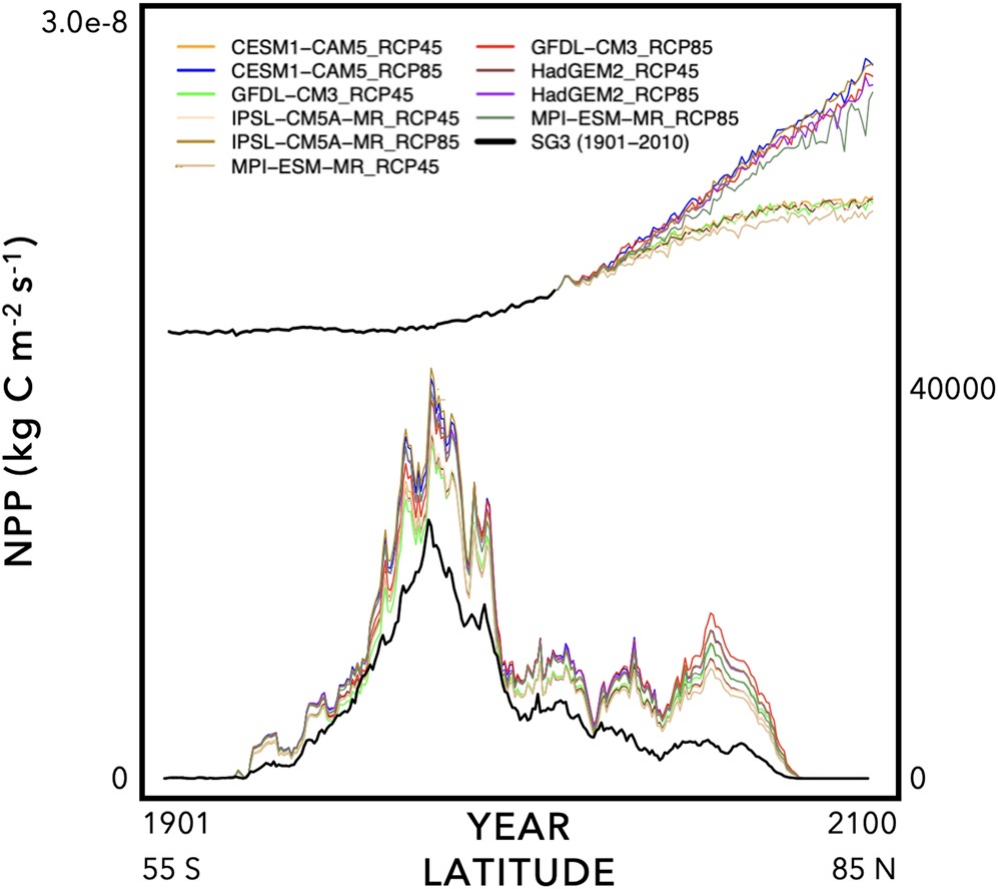
Inter‐simulation visual diagnostics for MsTMIP Phase II experiments show how a single model changes output among five different climate projections and two Representative Concentration Pathways (RCPs). The climate projections are from five Earth System Models: (1) CESM1‐CAM5, (2) GFDL‐CM3, (3) HadGEM2, (4) IPSL‐CM5A‐MR, and (5) MPI‐ESM‐MR. The two RCPs are: (1) business‐as‐usual (RCP8.5) and (2) medium mitigation (RCP4.5). Output is for Net Primary Productivity (NPP) for the HyLand model. The top set of lines (primary *y*‐axis) is the annual average from 1901 to 2100 (1901–2010 is from the retrospective SG3 scenario, black line). The bottom set of lines (secondary *y*‐axis) is the latitudinal/zonal sum from 55°S to 85°N.

An additional set of diagnostic visualizations are produced from the Experiment package to compare among different model experiments for a single given model (Figures 4 and 5). This is to ensure that there was indeed a difference among experiments, which may not happen if there was a problem in setting up the experiment. Moreover, this is also to ensure that the difference between model experiments coincides with theoretical expectations, for example, increasing NPP with increasing CO_2_ (Schimel et al., 2015; Walker et al., 2021). Figures 4 and 5 are separated because the magnitude difference between Phase I experiments (Figure 4) and the output from Phase II (Figure 5) is so large that the Phase I differences would be too small to see on the raw Phase II scale. For the inter‐simulation runs, we find that line plots are more useful for diagnosis than are maps, as differences and trends are more readily noticeable to the eye than in maps.

Numerous analyses have been published on 1901–2010 model and simulation experiment intercomparisons from MsTMIP and other MIPs (e.g., Cui et al., 2019; El Masri et al., 2019; Fang et al., 2017; Fisher et al., 2016; He et al., 2020; K. Huang et al., 2018; Huntzinger et al., 2017, 2020; Ito et al., 2016; Kolus et al., 2019; Liu et al., 2019; Mao et al., 2015; Schwalm et al., 2015, 2017, 2019, 2020; Shao et al., 2016; Thomas et al., 2016; S. Zhou et al., 2017, 2018). However, the 2011–2100 Phase II output is novel with multiple land models having been run with a suite of climate and RCP projections (see, also, Warszawski et al., 2014). In the annual time series example in Figure 5, we can see simultaneously the spread in NPP caused by differences (or uncertainties) in climate, which turn out to be relatively small compared to the large differences due to CO_2_ scenario (RCP 4.5 vs. RCP 8.5). These results continue to suggest the overwhelming dominance of the CO_2_ fertilization effect uncertainty on land carbon uptake uncertainty both historically and into the future (Arora et al., 2020; Huntzinger et al., 2017; Schimel et al., 2015; Sitch et al., 2015; Walker et al., 2021).

## 4 Discussion

The Terrestrial Biosphere Model Farm aims to reduce the time and skill required to execute a model experiment. An undergraduate student with no modeling experience and moderate programming skills can integrate a new TBM, spin‐up the TBM, and execute at least one experiment with a 10‐week summer internship. Indeed, many of the coauthors on this paper were those undergraduate interns. The core technical team would already have the TBM available and compiled with supported libraries installed and debugged before those internships began. Interns would spend the first few weeks learning about TBMs in general, studying the model code, and mapping the model I/O structure. They would then spend at least a couple of weeks mapping the LCLUC forcing data onto the TBM PFT structure. Another week or two would be spent connecting the model to the Farm and running tests. The next couple of weeks would be spent on spin‐up and debugging. The final couple of weeks could allow the first MsTMIP simulation, assuming all else was successful to that point (which was not always). Some interns continued working on their model into their school year or returned for a following summer for more model runs and experiments. These examples illustrate the relative ease and challenges that the Farm afforded for running TBMs in contrast to, for example, traditional model development centers.

Generally, the Terrestrial Biosphere Model Farm provided several complements to traditional MIPs. Once the models were properly setup, it was relatively fast to run multiple experiments across models. Moreover, a team organizing a MIP might be interested in many different types of experiments—more than they would be comfortable specifying not wanting to bother so many people and risking incomplete runs. With a centralized Farm, once the forcing data and experiments are setup, multiple models can run with relative ease. One example of different types of experiments that the Farm supported was on LCLUC data set development for NASA's Carbon Monitoring System (CMS) (Kennedy et al., 2018; Neeti & Kennedy, 2016; Y. Zhou et al., 2021). Here, the team focused on new and different types of LCLUC data products and their impacts on TBMs. We were able to ingest these different LCLUC data sets and provide the corresponding variability in TBM output. Ultimately, future MIPs should expand to include not only multiple models, but also multiple forcing data sets (Dietze et al., 2014). Additionally, the Farm can be used as a "sandbox" with which to test and debug any problems with the new experiments. Once those are worked out, then the MIP can be extended to individual modeling teams so that each team does not have to waste time working out those bugs.

For each model, there were a number of user decisions that had to be made in setup, for example, how to map the LCLUC forcing data onto the model PFTs. For instance, a pixel may be classified as “mixed forest” in the forcing data, but a model may have multiple forest PFTs (Poulter et al., 2011). By erring consistently toward a single definition of mixed forest, for better or for worse, we could at least constrain differences in model output due to those types of user decisions. A similar process may be considered for classifying different crop types. Still, LCLUC mapping and debugging was a very time‐consuming process. Likewise, while MIPs may specify spin‐up requirements and forcing data, it is challenging to know if the modeling teams adhered completely to those specifications. In the Farm, all models were run uniformly across those specifications, even if models had different ways of going about spin‐up, time steps, and use of data. However, while these aspects potentially reduce spread among model outputs, we also saw ways in which one could tune models to match benchmark data sets (e.g., scalars for stocks and fluxes). Being model and output agnostic, we did not tune. But, as noted earlier, models may converge toward each other in traditional MIPs simply because of tuning (Dommenget & Rezny, 2018; Fer et al., 2021; Hourdin et al., 2017; Notz, 2015; Raäisaänen, 2007; Scheiter et al., 2013).

Nonetheless, there were challenges in running the Farm and using its output. While “supercomputers” may be super at fast processing, they can be bottlenecked by file storage and transfer (Xie et al., 2012). Multiple models and multiple scenarios for hundreds of years for thousands of pixels result in a lot of data. Supercomputers are not data archive centers, and so a conflict arises when these requirements are levied upon them at least in the relative short term. There needs to be a place to move intermediary model output before they are finalized for storage at a data archive, allowing time for debugging and output assessment. Moreover, supercomputers are a shared resource, and one cannot monopolize time on them for extended periods. Patches that require extra spin‐up time to reach equilibrium run into that conflict, requiring disproportionate labor to complete a small number of pixels. Additionally, supercomputers are continually evolving, updating, and being replaced (Lim et al., 2015). Migration and re‐setup of the Farm are very time‐consuming for each new system.

Beyond the technical challenges, there are bounds to the scientific utility of the Model Farm output. Like supercomputers, individual models are continuously evolving and updated by the respective modeling teams (e.g., Bonan, 1998; Bonan & Levis, 2006; Bonan et al., 2002; Lawrence et al., 2011, 2019). It requires significant labor to repeatedly replace Farm models with their most recent versions; inevitably, the models in the Farm may not be up to date. As such, examination of model Farm output may not necessarily be from the most recent model versions. This issue is common for traditional MIPs too, though modelers sometimes update their results later for the MIPs as their models change. Some MIPs manage to conduct regular updates (Friedlingstein et al., 2020). On the other hand, models in the Farm might be considered more stable than newer model versions that may still have bugs to be discovered. Further, when trying to publish results from most MIPs, reviewers typically hit those papers with, “Why did the models behave the way they did?” This is challenging for any individual modeler to answer about their own model, let alone a centralized system that runs other people's models as effective “black boxes.”

So, how do we overcome these challenges, yet still advance the needs for robust MIPs? In Fer et al. (2021), we presented a roadmap to community cyberinfrastructure for ecological data‐model integration. Five community tools are needed to accelerate the merging of models and data: (a) shared workflows, (b) scalable data ingest, (c) calibration, (d) benchmarking, and (e) iterative data assimilation. While the Farm addresses (a), (b), and some of (d), all five of these tools are implemented within a multimodel workflow management system called PEcAn (Dietze et al., 2013; LeBauer et al., 2013). PEcAn, like the Model Farm, is a tool to run multiple TBMs on a centralized system. However, unlike the Model Farm, PEcAn is an open‐source utility that is available to anyone (pecanproject.org), can be installed on anything from a laptop to a supercomputer, or can be run in the cloud. As such, PEcAn operates as a distributed network of nodes rather than a single centralized server, which allows MIPs to either be fully centralized on one node—analogous to the Farm—or run by individual teams and synced via the PEcAn API, which may reduce the “other people's models” challenge mentioned earlier. Because PEcAn is containerized in Docker, it also greatly reduces the challenges of getting new models compiled and running on new serves as well migrating the system when systems are upgraded.

PEcAn provides a number of language‐agnostic API tools to assist with model calibration, data fusion, analysis, and provenance tracking (Fer et al., 2018; LeBauer et al., 2013; Shiklomanov et al., 2020). As part of its data assimilation workflow, PEcAN can quantify, propagate, and analyze uncertainties associated with parameter, driver, and initial condition uncertainty distributions (Dietze et al., 2014). Further capabilities are in progress that are not too dissimilar from a community model including, for example, links to benchmarking and validation efforts (Abramowitz, 2012; Blyth et al., 2011; Collier et al., 2016; Kelley et al., 2013; Luo et al., 2012; Randerson et al., 2009; Schwalm et al., 2010). PEcAn's initial development has been focused on site‐ to continental‐scale research; whereas, the Model Farm has focused on global runs, requiring the distribution and gathering of inputs and outputs. Still, like the Farm, PEcAn requires significant modeler time to develop couplers for inputs, runs, and updates. Nonetheless, PEcAn provides an excellent platform for unifying models for MIPs and ultimately saves researchers' time on developing the informatics and analysis tools built into PEcAn; we highly recommend that modelers begin to explore it. A blend of a centralized Farm team with the open platform of PEcAN could be a beneficial combination. The next frontier is a merger of the strengths of the Model Farm and those of PEcAn, which can create a whole that is larger than the sum of its parts.

## 5 Conclusion

**Figure 6:**
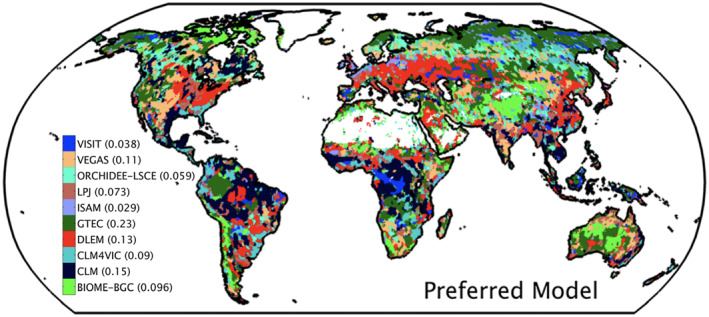
The terrestrial Earth as represented by the top‐performing terrestrial biosphere model in MsTMIP based on a suite of benchmarks. From Schwalm et al. (2015).

Earth system modelers are like the blind men and the elephant, each trying to seek the truth in front of them—the future of the Earth; and, each saying something different, yet similar. By working together through MIPs, we may be able to piece that picture together (Figure 6). Yet, MIPs are challenging, and we need to ensure that each of those voices accurately depicts a defined perspective. We attempted to systematically unify and control for a number of “free parameters,” or degrees of freedom, in MIPs through a centralized Model Farm. While solving these problems raised other challenges, we did bring something novel to the conversation of how MIPs are operated and interpreted. As we collectively continue to strive toward understanding the future, we must not only continue working together, but we must do so with improved consistency, transparency, and efficiency. And, we must do so quickly, as time is of the essence—the elephant may be gone by the time we figure out what was in front of us.

## Conflict of Interest

The authors declare no conflicts of interest relevant to this study.

## Data Availability

The authors primarily used the MsTMIP data and simulation protocols (Huntzinger et al., 2013; Wei et al., 2014).
